# DNA Damage: From Threat to Treatment

**DOI:** 10.3390/cells9071665

**Published:** 2020-07-10

**Authors:** Antonio Carusillo, Claudio Mussolino

**Affiliations:** 1 Institute for Transfusion Medicine and Gene Therapy, Medical Center—University of Freiburg, 79106 Freiburg, Germany; antonio.carusillo@uniklinik-freiburg.de; 2 Center for Chronic Immunodeficiency, Faculty of Medicine, University of Freiburg, 79106 Freiburg, Germany

**Keywords:** genome integrity, DNA damage, DNA damage response (DDR), cell-cycle, NHEJ, HDR, cancer, gene editing, CRISPR-Cas

## Abstract

DNA is the source of genetic information, and preserving its integrity is essential in order to sustain life. The genome is continuously threatened by different types of DNA lesions, such as abasic sites, mismatches, interstrand crosslinks, or single-stranded and double-stranded breaks. As a consequence, cells have evolved specialized DNA damage response (DDR) mechanisms to sustain genome integrity. By orchestrating multilayer signaling cascades specific for the type of lesion that occurred, the DDR ensures that genetic information is preserved overtime. In the last decades, DNA repair mechanisms have been thoroughly investigated to untangle these complex networks of pathways and processes. As a result, key factors have been identified that control and coordinate DDR circuits in time and space. In the first part of this review, we describe the critical processes encompassing DNA damage sensing and resolution. In the second part, we illustrate the consequences of partial or complete failure of the DNA repair machinery. Lastly, we will report examples in which this knowledge has been instrumental to develop novel therapies based on genome editing technologies, such as CRISPR-Cas.

## 1. DNA Lesions as a Constant Threat to the Cell

**Figure 1:**
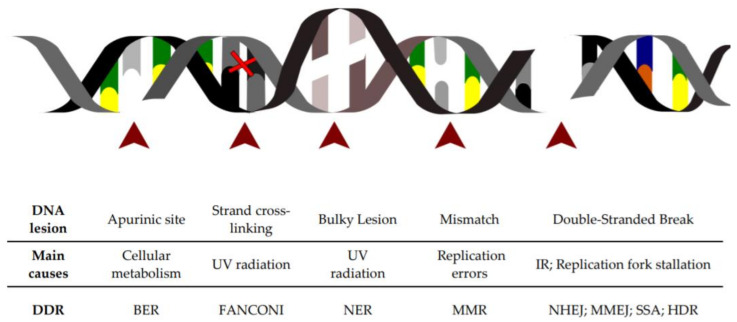
Schematic of the major DNA lesions experienced by cellular genomic DNA: The table below indicates the type of DNA lesion depicted in the figure above, its leading cause and the DNA repair pathway engaged for its resolution.

DNA harbors the genetic information necessary to build an organism, and its maintenance is pivotal for sustaining life. In order to ensure its faithful replication and transfer to daughter cells, multiple mechanisms have evolved across the centuries to detect and counteract possible DNA lesions that may threat genome integrity (Figure 1) [1]. Each human cell experiences more than 10,000 DNA lesions per day, most of which are typically caused by normal cellular processes [2]. For example, spontaneous errors during the replication of DNA might result in the incorporation of wrong nucleotides in the newly synthesized DNA molecule, causing mismatched base pairing [3]. Similarly, Reactive Oxygen Species (ROS) or Reactive Nitrogen Species (RNS) are generally produced as byproducts of multiple physiological activities in diverse subcellular sites [4]. These chemical compounds are responsible for a variety of DNA lesions derived from oxidative stress, including the generation of apurinic/apyrimidinic sites (AP), single- or double-stranded breaks, and base substitutions [5]. Both ROS and RNS are considered an endogenous source of DNA-damaging agents. However, also exogenous sources, such as ionizing radiations (i.e., X-rays), cosmic radiation, mutagenic chemicals (like polycyclic aromatic hydrocarbons, a common byproduct in tobacco smoke) and ultraviolet (UV) light contribute to the load of DNA lesions that a cell has to counteract daily [6,7]. As all natural compounds, DNA also undergoes natural decay processes, such as alkylation, oxidation and deamination, which are mutagenic if not repaired as they lead to incorrect base pairing with consequent base substitutions during DNA replication [8]. Another common type of DNA lesion is the single-stranded break (SSB), which may occur spontaneously, for example, during DNA replication, fork stalling or collision of the transcriptional machinery [9], or as a consequence of UV or gamma irradiation [10]. Accumulation of SSBs or the occurrence of multiple SSBs in close proximity may lead to complex lesions, where both strands of the DNA are severed, and so-called double-stranded breaks (DSBs) which are one of the most dangerous DNA lesions that the cell can experience [10]. Hence, genetic information and genomic stability are constantly threatened by multiple causes and the cells have evolved complex mechanisms to sense, monitor and repair this wide variety of DNA lesions. 

## 2. The DNA Damage Response Is Activated upon Sensing of DNA Lesions

The DNA Damage Response (DDR) can be considered an endogenous alarm system constantly monitoring the genome integrity and ensuring the faithful transmission of the genetic information to daughter cells. Each DNA lesion described above leads to specific DNA alterations for which the cells have evolved lesion-specific repair mechanisms (Figure 1). Here, we provide a simple overview of these mechanisms. For a comprehensive description of the complex pathways involved, we refer the reader to extensive review articles previously published [11,12,13]. Interstrand crosslinks (ICLs), generated either by natural or synthetic compounds, are mostly resolved via the Fanconi Anemia (FA) pathway [14]. An increasing body of evidence suggests that ICLs are recognized by dedicated proteins, such as ubiquitin-like containing plant homeodomain, RING finger domains 1 (UHRF1) and UHRF2 that interact and facilitate the recruitment and retention of Fanconi anemia group D2 (FANCD2) protein at the ICL site [15]. This event is central to the function of the FA pathway, and FANCD2 posttranslational modifications are essential for proper regulation of this DNA repair pathway and subsequent resolution of ICLs [14]. Bulky DNA lesions, as a consequence of UV irradiation, environmental mutagens and certain chemotherapeutics are sensed by the xeroderma pigmentosum group C (XPC) protein that activates the Nucleotide Excision Repair (NER) pathway. On the other hand, alkylated, oxidized and deaminated bases, resulting from natural DNA decay or endogenous DNA damaging agents, are recognized by specific DNA glycosylases that activate the Base Excision Repair (BER) pathway. In case of mismatched bases incorporated during DNA replication, the MutS Homolog (MSH) heterodimer complexes lead to the activation of the Mismatch Repair (MMR) pathway that secures the resolution of the lesion. If SSBs form, typically because of oxidative stress or as intermediate products of different DNA repair pathways, they are sensed via the Poly(ADP-ribose)polymerase 1 (PARP-1) complex and usually resolved via a pathway similar to the BER pathway [16]. When a DSB occurs, phosphorylation of the H2A histone variant X (termed γH2AX), mostly at Serine 139 [17], is one of the first events priming the DDR signaling cascade. The γH2AX histone decorates the DSB site and acts as a signaling beacon, marking genomic lesion. Phosphorylation of H2AX is promoted by the phosphatidylinositol 3-OH-kinase-like family including ATM (ataxia-telangiectasia mutated), DNA-PKcs (DNA-dependent protein kinase catalytic subunit) and ATR (ataxia telangiectasia and Rad3-related), which are all activated in response to DSB [17] or replicative fork stalling respectively [18]. 

The exact cascade of events leading to sensing of a DSB is still debated. The most accredited model as of today suggests that changes in chromatin architecture, as a result of a DSBs, are sensed by ATM. This results in its activation via the autophosphorylation of Serines S367, S1893, S1981 and S2996 and eventually leads to ATM localization at the DSB site [17]. The subsequent phosphorylation of H2AX recruits MDC1 (mediator of DNA damage checkpoint protein 1) that functions as docking site for the MRE11-RAD50-NBS1 (MRN) complex [19]. The signaling is reinforced by further ATM deposition promoted by MRN, and the recruitment of additional DNA repair factors eventually leads to the formation of the so-called Ionizing Radiation Induced Foci (IRIF) [20]. To provide the cells enough time to repair the DNA lesion, ATM is also capable of modulating the function of the checkpoint kinases 1 and 2 (CHK1 and CHK2) and of slowing down the cell cycle [21], as discussed in the next section. However, when the DSBs cannot be repaired, the unresolved DDR signaling triggers cell senescence or apoptosis via p53-induced cell-cycle arrest. This fascinating mechanism is extensively reviewed elsewhere [22]. 

## 3. DNA Damage and Cell Cycle Checkpoints 

The cell cycle is divided into four main phases, and internal checkpoints guarantee normal progression if the necessary condition for cellular growth and division are met [23]. Although the checkpoints are distinct, they all respond to lesions in DNA and, partially, some of the effector proteins are shared among the different checkpoints [24]. Cell cycle progression is mostly orchestrated by the cyclin-dependent kinases (CDKs), a family of protein kinases that phosphorylate key substrates to promote DNA synthesis and cell cycle progression [25]. In mammals, the CDK family is composed of more than 20 members acting at different cell cycle phase transitions such as CDK2 and CDK4 that regulate G1–S transition [26,27] or CDK1 and CDK2 that control G2–M transition instead [28]. Cross-talk between DDR and cell cycle progression secures that the latter is modulated to either favor the repair of dangerous DNA lesions or to induce senescence or cell death when repair fails [29,30]. When DNA damage is sensed during the G1 phase, the activity of the transcription factor p53 is critical before entry into the S phase. Upon its activation and stabilization, p53 is capable of driving the expression of a large number of genes, including the cyclin-dependent kinase inhibitor p21 (CDKN1A), leading to G1 arrest [31]. While the cell cycle is halted, the driving DNA lesion is removed by the specific DDR mechanism harnessed prior to DNA replication. A contribution to the G1-S checkpoint comes also from the p38 mitogen-activated protein kinases (MAPK) pathway, which is activated in response to a wide range of DNA damage events [32]. Particularly, upon DSB formation, p38 MAPK is activated by ATM through the Tao (thousand and one) kinases [33]. Upon activation, p38 MAPK is able to directly activate p53, leading to the accumulation of p21. This protein counteracts CDK2 activation by directly inhibiting its cyclin interactors (cyclin D and CDC25A) [34]. Additionally, p38 MAPK is also able to directly phosphorylate and stabilize p21, further promoting G1 arrest [35]. Importantly, p21 levels are crucial to promote G1 arrest, and only when a certain threshold is reached, the block occurs, thus preventing premature cell-cycle arrest [36]. Once the threat has been resolved, the cell cycle can proceed to the S phase, during which synthesis and replication of DNA occurs. During this phase, the sensing of DNA damage typically results in slowing down rather than halting of DNA synthesis in order to accommodate but not necessarily repair the lesion [37]. The biological significance of this checkpoint is to provide the repair machinery with sufficient time to resolve aberrant DNA structures and to prevent cells from dividing before their entire genomes are faithfully duplicated [38]. This is possible through the activation of multiple origins of replication which have been established early during cell cycle and can be activated if necessary by the concerted action of ATM and ATR [39]. Once the G2 phase of the cell cycle is initiated, an additional checkpoint secures that the DNA is completely replicated prior to mitosis. In this case, cell-cycle progression is regulated by the accumulation of inactive CDK1. This leads to an excess of non-phosphorylated retinoblastoma (Rb) tumor suppressor, which is able to inhibit cell-cycle progression [40]. Another key player in G2 arrest is p21 that is able to directly block Rb phosphorylation and to sequester inactive CDK1 in the nucleus, inhibiting entry to the mitotic phase [40]. In conclusion, despite the DNA lesion that triggers DDR, the cell has developed unique mechanisms to halt or slow down the progression of the cell cycle to allow repair of the injury. Importantly, besides the type of DNA lesion, also the phase of the cell cycle during which DNA damage is sensed dictates which DNA repair pathway will be engaged [41]. A detailed explanation of the different pathways that secure seamless replication of the genomic DNA will be given in the next section.

## 4. DNA Repair Pathways

As described in the sections above, cells possess surveillance systems able to recognize DNA damage, allowing a proper response according to the type of lesion experienced. Upon DNA damage detection, information is transferred to the mechanisms managing the cell cycle progression in order to pause or slow down cell division. This in turn activates a state during which the cell can adopt the repair mechanism suitable to repair the lesion. In this section, we will describe the mechanisms activated by the cell to endure and resolve DNA damage. 

### 4.1. Fanconi Anemia (FA) Pathway

Several natural or artificial compounds might promote crosslinking of two DNA strands. This form of DNA damage, typically referred to as interstrand crosslink (ICL), is highly dangerous as it prevents transcription and replication of DNA by inhibiting strand separation, leading to p53-dependent apoptosis [42]. Studies aimed at better understanding the mechanisms leading to a severe genetic disorder, known as Fanconi anaemia (FA), have shed light on how cells react and repair this DNA lesion. Indeed, typically, FA patients suffer from high sensitivity to ICL-forming agents. For a detailed description of this complex DNA repair pathway, we refer the reader to a comprehensive review previously published [14]. In the majority of cases, ICL recognition occurs during DNA replication and involves UHRF1, which in turn recruits FANCD2 at the damaged site. The latter, together with Fanconi anemia group I protein (FANCI), orchestrates the complex pathway, leading to ICL resolution. Ubiquitination of the FANCD2/FANCI complex promotes the recruitment of endonucleases, such as DNA excision repair protein (ERCC1) and Xeroderma Pigmentosum group F (XPF), that cleave the DNA and unhook the lesion [43]. The resulting DSB is then repaired via the concerted activity of exonucleases, polymerases and ligases and is detailed elsewhere [42]. 

### 4.2. Nucleotide Excision Repair (NER)

In eukaryotes, NER can be divided in two distinct pathways, the global-genome and the transcription-coupled NER extensively reviewed previously [44]. These two sub-pathways differ only in the mechanism by which they recognize the DNA lesion. In GG-NER, bulky DNA lesions lead to a major distortion of the DNA helix and are recognized by the XPC protein, which is in turn stabilized by RAD23B [45]. On the other hand, TC-NER is independent from XPC, but it occurs when RNA polymerase II stalls at a DNA lesion serving as a signal for damage recognition. Once the DNA lesion is recognized, the 10-subunit complex of transcription factor IIH (TFIIH) is recruited. This comprises the XPD (Xeroderma Pigmentosum group D) helicase, which unwinds the DNA helix, and two other members of the complex, XPA (xeroderma pigmentosum group A) and replication protein A (RPA), which stabilize the DNA. Subsequently, the endonucleases within the complex, namely XPG (Xeroderma Pigmentosum group G) and XPF (Xeroderma Pigmentosum group F), cleave the damaged DNA strand at both sides of the lesion and remove it. Subsequently, the gap is filled by the concerted action of delta/kappa and epsilon polymerases, along with DNA ligase IIIα or DNA ligase I [46].

### 4.3. Base Excision Repair (BER)

As mentioned previously, natural DNA decay or endogenous DNA-damaging agents can cause small alterations of the DNA helix. These lesions are recognized by one of the eleven known damage-specific DNA glycosylases, which in turn activate the BER pathway [47]. In the first phase, the glycosylase removes the aberrant base by cleaving the N-glycosylic bond between the base and the deoxyribose and generates an abasic site (AP). Depending on the length of the gap, further processing occurs via distinct mechanisms, namely short-patch (for single nucleotide processing) or long-patch (for 2–10 nucleotide processing) BER. The AP is sensed and bound by the PARP-1 and its homolog PARP-2, which recruit the human AP-endonuclease 1 (APE1). This protein nicks 5′ of the AP, generating proper 3′-hydroxyl and 5′-deoxyribose phosphate termini in which the DNA polymerase beta can insert the missing nucleotide(s). In short-patch BER, the gap is finally sealed by the concerted action of the X-ray repair cross-complimenting protein 1 (XRCC1), DNA ligase I and DNA ligase III. In long-patch BER, gap sealing is mediated by proliferating cell nuclear antigen (PCNA) [47,48]. When AP sites are not properly resolved, they might lead to a peculiar type of DNA lesion, the SSB. These lesions are fixed via a pathway similar to the BER. Indeed, SSB detection is mediated by PARP-1, which recruits the APE2 endonuclease to the SSB site. This enzyme unmasks a longer single-stranded DNA portion. which is in turn recognized by a complex cascade of different proteins that eventually recruit polymerases and ligases to seal the gap [9].

### 4.4. Mismatch Repair (MMR)

DNA mismatch repair (MMR) is a highly conserved DNA repair system that critically contributes to maintaining genome stability through the correction of mismatched base pairs. Mismatches primarily occur as errors during DNA replication, although they can arise also from other biological processes [12]. Mismatched bases are recognized by two main MutS Homolog (MSH) heterodimer complexes: MutS alpha, which comprises the MSH2 and MSH6 subunits, and MutS beta, including the MSH2 and MSH3 subunits [49]. The former mainly identifies single base pair mismatches and small insertion/deletion (indel) mutations of one or two bases. while the latter mediates the recognition of larger indels [50]. Mismatch recognition by MutS alpha and beta complexes causes a conformational change of the complex itself into a sliding clamp which primes the recruitment of an exonuclease, probably EXO1, which leads to the removal of about 150 nucleotides surrounding the mismatched region. The gap is then filled by the DNA polymerase delta and DNA ligase I [49].

### 4.5. DNA Double-Stranded Break Repair Pathways

**Figure 2:**
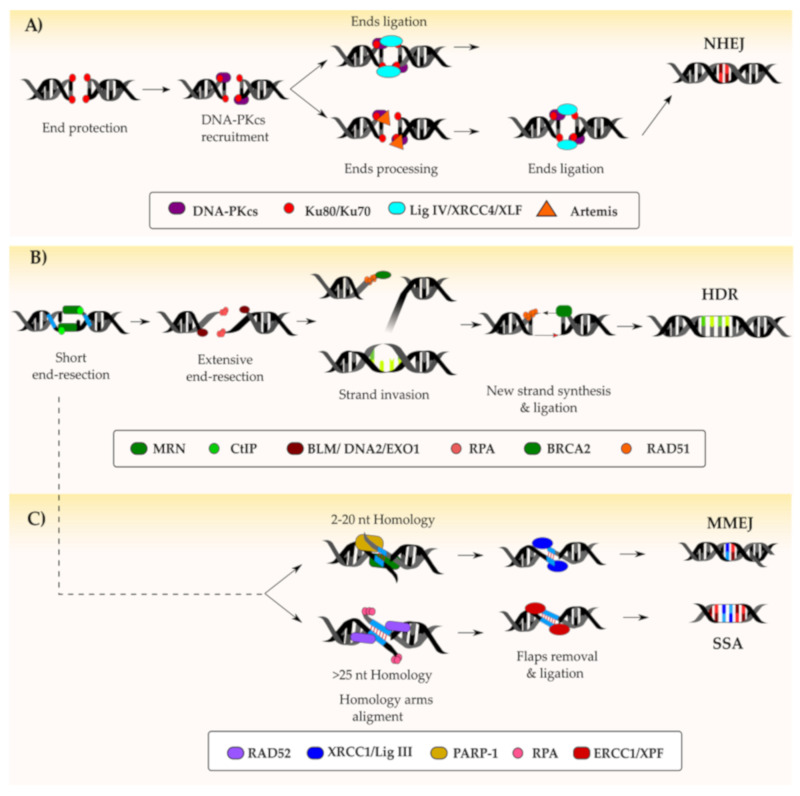
Resolution of a DNA double-stranded break (DSB): Following the recognition and marking of a DSB by the concerted action of key proteins such as ATM (ataxia-telangiectasia mutated), ATR (ataxia telangiectasia and Rad3-related) and DNA-PKcs (DNA-dependent protein kinase catalytic subunit), the repair can follow different pathways. On one hand, if the DNA ends are protected by the Ku70/Ku80 complex, the DSB is generally repaired via the non-homologous end-joining (NHEJ) pathway (depicted in **A**). In this case, an intermediate end-processing step might be necessary if the protected ends are not compatible. On the other hand, resection of the DNA ends by the MRN/CtIP (MRE11-RAD50-NBS1/C-terminal-binding protein interacting protein) complex results in NHEJ inhibition. Based on the length of homology DNA fragments revealed during end-resection, the DSB can be repaired either via homology-directed repair (HDR) (**B**) or microhomology-mediated end joining (MMEJ)/ single-strand annealing (SSA) (**C**). The key factors involved in the DNA repair pathways depicted are indicated within the figure.

DSB is one of the most critical and dangerous types of DNA lesions leading, if not repaired, to cell death. Mammalian cells have evolved multiple mechanisms that contribute to DSB repair (Figure 2). In general, these mechanisms can be divided into conservative, characterized by the accurate repair of the DSB, and nonconservative that might lead to the alteration of the original DNA sequence. The cell cycle phase during which the lesion occurs and the extent of resection that the broken DNA ends undergo contribute to the type of repair mechanism engaged [51]. Multiple mechanisms are involved in the repair of DSBs, and details are given in the following paragraphs. 

#### 4.5.1. Non-Homologous End-Joining

Non-homologous end-joining (NHEJ) is the pathway preferentially adopted by the mammalian cells to resolve DSBs (Figure 2A). As mentioned in the second paragraph of this review, DSBs are signaled through the phosphorylation of the H2AX histone variant which activates the cellular DDR machinery. The first component of the NHEJ pathway recruited at the DSB is the Ku70-Ku80 dimer. This binds to the two ends of the DNA break and protects them from end-resection, thus antagonizing the HDR pathway [52]. The C-terminal domain of Ku80 promotes the recruitment of the DNA-PKcs to form the DNA-PK complex [53]. DNA-PK can recruit the X-Ray repair cross-complementing protein 4 (XRCC4), XRCC4-like factor (XLF) and the DNA ligase IV to seal the break if the ends are compatible [54,55,56]. If the ends are not compatible, DNA-PKcs undergoes self-phosphorylation at the so-called ABCDE cluster and promotes the activation of the Artemis nuclease [57]. This, in a complex with DNA-PKcs, resects the DNA ends which are then stabilized by XRCC4 and XLF prior to their ligation operated by the DNA ligase IV [54], with the contribution of the recently discovered paralogue of XRCC4 and XLF (PAXX) [58]. In case of chemistry of the DNA ends not supporting direct ligation, additional modifications might be required. Editing of aberrant ends, either lacking a 5′-phosphate or having a 3′-phosphate, is typically processed by the polynucleotide kinase (PNK), which possesses both kinase and phosphatase activity [59]. The eventual ligation step of the two DNA ends is further enhanced by the activity of polymerases that support the DNA ligase IV activity when ends are not compatible. For example, the template-independent Polµ promotes the rejoining of noncompatible 3′-overhangs by generating microhomology stretches at the DNA ends [60]. On the other end, compatible ends that still require the fill in of missing nucleotides are processed by Polγ in a template-dependent manner. Even though the DNA-PK complex is mostly involved in promoting NHEJ-mediated repair, it can also favor HDR. Indeed, when NHEJ is nonfunctional, the autophosphorylation of the ABCDE cluster releases the DNA ends from the Ku70-Ku80 dimer. This in turn results in HDR-promoting factors gaining access to the DNA ends, thus initiating the extensive DNA resection necessary to engage the HDR pathway [61]. In case of defective NHEJ, this alternative resolution ensures that DSBs are repaired prior to activation of senescence and apoptosis [62,63].

#### 4.5.2. Homology-Directed Repair

The homology-directed-repair (HDR) pathway promotes the scarless resolution of the DNA lesion by using the sister chromatid as a template (Figure 2B). In this case, the DSB is sensed by two MRN complexes that localize at both ends of the DSB [64] even though the mechanism by which MRN is able to get access to DNA ends that are protected by Ku70-Ku80 heterodimers is still elusive [65]. This complex contributes to both antagonizing NHEJ and to amplifying the γH2AX phosphorylation signal [64,66]. Subsequently, the two MRN complexes are tethered together in a process mediated by RAD50 [67]. However, to properly engage HDR, an extensive DNA end resection is necessary [52]. This is initiated by MRE11 that, through its endonuclease activity, induces a distal nick up to 300 nucleotides away from the DSB. Subsequently, its 3′–5′ exonuclease activity extends the nick back towards the DSB [68]. This first short resection is intended to displace the Ku complex and inhibits further processing by NHEJ factors [69]. Resection is extended with the contribution of C-terminal-binding protein interacting protein (CtIP) exonuclease, which is activated by phosphorylation at Serines S233, S276 and S347 [70]. Upon reaching the DSB, the CtIP dissociates and the resection is extended by Exonuclease 1 (EXO1) with the support of endonuclease DNA 2 and the Bloom syndrome helicase (BLM), which mediates DNA unwinding [70,71]. Although DNA end resection is critical for the harnessing of HDR, this process leads to cell death if not properly regulated. Indeed, when the HDR antagonist p53-binding protein 1 (53BP1) is absent, extensive and uncontrolled DNA end resection leads to deleterious chromosomic rearrangements and eventually to apoptosis [72,73]. To avoid excessive DNA processing, the exonuclease activity of EXO1 is limited by ATM-mediated phosphorylation or its proceeding is inhibited by physical barriers, such as the presence of 53BP1 [74]. The single strand DNA (ssDNA) generated during the end resection process is immediately recognized and bound by the Replication Protein A (RPA) complex (RPA1, RPA2 and RPA3), which also removes secondary structures formed during the process [75,76]. Next, the DNA Repair Protein RAD52 mediates the removal of RPA and allows RAD51 recombinase loading onto the 3′-end of the long ssDNA. In this process BRCA2 (Breast Cancer Type 2 Susceptibility Protein) plays a key role, too, both sustaining the displacement of RPA and the loading of RAD51 [77]. The formation of the RAD51–ssDNA nucleoprotein filament is crucial for the subsequent steps of HDR as it mediates the search for homologous sequences on the sister chromatid and formation of D-loop triple-helix structures, essential for transferring the genetic information to the new DNA strand [78,79]. Eventually, repair can be completed via mechanisms that lead to either crossover or non-crossover events as reviewed elsewhere [80]. 

#### 4.5.3. Alternative DSB Repair Pathways

DSBs in mammalian cells are typically repaired either via NHEJ or HDR. However, cells possess alternative pathways which are distinguishable when NHEJ is not properly functioning. These are classically referred to as alternative end-joining (A-EJ) or single-strand annealing (SSA; Figure 2C) and are detailed elsewhere [53,81]. Both A-EJ and SSA are error-prone as they involve the loss of nucleotides and require stretches of homologous sequences of different lengths on the same chromosome, which are used as a repair template: short homology stretches (i.e., 2–20 nucleotides) activate microhomology-mediated end joining (MMEJ), while longer stretches (i.e., >25 nucleotides) promote SSA [81]. As explained above, when the DSB is sensed, the first short resection step mediated by MRN and CtIP occurs. If short homologous DNA sequences are exposed on the two resected ends, PARP-1, MRN and polymerase theta mediate the alignment of the complementary DNA strands. The non-homologous 3′-flaps are removed, and the gaps are ligated by the XRCC1/ligase III complex. If homology regions longer than 25 nucleotides are unmasked, RPA protects the single-stranded DNA while RAD52 mediates the base pairing of homologous sequences. The non-homologous 3′-flaps are removed by the ERCC1/XPF endonuclease complex and the gap is filled by the coordinated action of DNA polymerases and DNA ligases [82]. 

## 5. DNA Repair Defects

### 5.1. Linking DDR Failure to Cell Disorders and Cancer

We have largely described the critical role that the DNA damage repair pathways have in the maintenance of genome integrity. It is therefore not surprising how defects in these mechanisms might be detrimental for cell physiology. Mutations might accumulate if specific DNA repair pathways and/or checkpoints operate aberrantly or if the DDR is overburdened. These events might generate first hits, leading to genome instability and malignant transformation. The Cancer Genome Project and Cancer Genome Atlas have analyzed more than nine thousand samples encompassing 33 different cancer types, providing a general signature of cancer associated aberrations. These mutations are typically divided into “driver”, that directly promotes cancer initiation and progression, and “passenger”, contributing to cancer development as a consequence of their accumulation [83]. Somatic mutations affecting DDR-related genes were found in about a third of the cases. Examples are represented by mutations in the *MLH1* and *MSH2* genes that belong to the MMR pathway. When these genes are mutated, the resulting dysfunctional MMR leads to failure in properly recognizing and resolving errors arising from physiological processes, such as DNA replication, therefore priming malignant outcomes [84] or predisposing to cancer [85]. However, alteration in DDR can trigger disorders other than cancer. For example, loss of protection against UV-mediated DNA damage resulting from inactivation of key players in NER is one of the causes leading to rare autosomal recessive diseases, such as xeroderma pigmentosum (XP), cockayne syndrome (CS) and trichothiodystrophy (TTD) [86]. Alterations in NHEJ have been associated with devastating immunologic and developmental defects [87]. While the majority of DSBs result from unwanted DNA lesion, immune cells harness this type of DNA damage to create diversity in crucial physiological processes such as V(D)J recombination, somatic-hyper-mutation (SHM) and class-switch recombination (CSR) [88]. These “programmed genomic alterations” are critical for the development of B and T lymphocytes during the generation of immunoglobulins (Ig) and T cell receptor (TCR) repertoire, respectively. Ig and TCR are made of variable regions which are shuffled and rejoined in various combinations to generate the variability necessary for recognition of multiple antigens. The mechanism by which shuffling is achieved comprises the activity of the RAG1/RAG2 complex that recognizes specific recombination signals flanking the DNA sequence of each V(D)J segment and introduces a nick at each site. Subsequently, each nick reacts with the opposite strand, generating the so-called covalently sealed hairpins at the two sites resulting in a DSB. The intervening sequence containing the recombination signals circularizes and is eventually lost during cell division. The two hairpins are then opened by the Artemis nuclease, upon its activation through the phosphorylation mediated by DNA-PKcs, and are sealed via the NHEJ machinery [89]. Therefore, defects in NHEJ factors critical for V(D)J recombination, such as Artemis, DNA-PKcs or LIG4, might lead to partial or complete absence of specific immune cells, resulting in a broad spectrum of immunodeficiencies, including severe combined immunodeficiency (SCID) [90]. As seen for NHEJ, inherited defects in HDR are also pathologic. Mutations in the *BRCA1* and *BRCA2* genes have been associated with predisposition to various cancers, including malignancies affecting breast tissue or ovaries, and with lower frequency in the prostate or pancreas [91,92]. Recently, other HDR-related genes have been associated with carcinogenesis when mutated, such as *PALB2* [93,94] and *CtIP* [95]. These multiple examples clearly show that failures in DDR can fuel and sustain cancer progression. On a positive note, many current cancer therapies, including radiotherapy and chemotherapy, exploit the failure of tumor cells to respond properly to DNA damage by inducing DNA lesions that prompt senescence. 

### 5.2. Exploiting Defects in DNA Repair to Treat Cancer

The main goal of cancer therapy is achieving complete elimination of the tumor either through surgical procedures or via the more or less selective killing of cancerous cells. Multiple strategies have been devised that target metabolic processes which are altered in cancer cells. Transformed cells are typically characterized by an extraordinary high replication rate. The use of antimetabolites, such as 5-fluorouracil (5-FU) or thiopurines, has been explored to inhibit nucleotides biosynthesis, thus depleting cells of the essential components to replicate their DNA and to proliferate [96]. Similarly, cell replication can be hampered by inhibiting the topoisomerase enzyme, which is essential to resolve DNA torsional stress occurring during replication. As a consequence, accumulation of DSBs and supercoiled structures before the replication fork limits cancer cell proliferation [97]. Since defects in DNA repair pathways are a fairly common feature in cancer cells, in principle, these cells are more vulnerable to DNA-damaging agents. The use of drugs to inhibit the remaining functional DNA repair pathways, an approach termed “synthetic lethality”, is often exploited to selectively kill the malignant cells [98]. A similar principle makes use of cancer cell vulnerability to oxidative stress for eradicating malignant cells. The high replication rate of tumor cells results in high oxidative stress, and cancer cells are highly dependent on pathways that prevent DNA damage under these circumstances [99]. Inhibitors of polymerase beta (POLB), a major factor in the BER pathway, have been exploited to drive cancer cell death [100]. However modest success and concerns related to inhibition of other polymerases has limited this approach [101]. Another common characteristic of tumor cells is a dysfunctional HDR repair pathway. Also in these cases, the use of agents that lead to the formation of DSB is typically explored to create overt DNA damage in cancer cells, leading to their death [102,103]. For instance, PARP-1 inhibitors have been widely used to inhibit SSB repair and to promote DSB formation in ovarian or prostate cancer [104,105]. Another mechanism to induce DNA damage makes use of cisplatin, which reacts with nucleophilic centers on purine bases, causing intra- and interstrand crosslink. This in turn leads to critical distortions of the DNA double helix and, if not resolved, to apoptosis [14]. Interestingly, combination of the PARP-1 inhibitor Niraparib with cisplatin has been used to overcome the acquired platinum-resistances of cancer cells that typically arises in some cases of metastatic ovarian cancer [106,107]. Although the potential of “synthetic lethality” strategies to combat some forms of tumors is unquestioned, in some cases. it fails due to the presence of a few tumor cells capable of counteracting the mode of action of the used compound. For example, BRCA1-deficient tumors treated with the PARP inhibitor Olaparib developed drug resistance in vivo in a murine model of BRCA1-associated breast cancer [106]. Further elucidation of the underlying mechanism revealed an upregulation of the *Abcb1A/B* gene encoding for the corresponding drug efflux transporters. This led to drug clearance, and the effect could be reversed by using Tariquidar, a drug that specifically blocks Abcb1 transporter activity [106]. Different drug resistance strategies have been reported that hamper the activity of PARP inhibitors. The use of Olaparib for breast cancer patients has been associated with acquired resistance to this inhibitor. This is achieved for example through the partial restoration of HDR as a result of reversion mutations in the *BRCA1* gene [108]. Although escape mechanisms exploited by cancer cells might dampen the full efficacy of “synthetic lethality”, they also offer the opportunity to reveal backup strategies adopted by tumors for survival. Further studies are necessary to generate novel therapeutics that can be rationally combined to neutralize multiple pathways at once. Ongoing studies on large patient cohorts will certainly contribute to improving our knowledge of escape mechanisms and to developing new strategies to counteract tumor progression. 

## 6. Exploiting DSB Repair to Develop Innovative Therapeutic Strategies

In the last decades, understanding the genetic bases of many human disorders has made unprecedented steps forward. This has fostered the development of novel therapeutics aimed at curing patients affected by genetic defects by means of gene therapy. This typically includes the development of viral vectors transferring a correct copy of the mutated gene. Once present in the host cell, the expression of the correct gene complements the missing gene function in the patient cells, resulting in a functional cure [109]. With the dawn of targeted genome editing, the chase for new approaches aimed at precisely correcting the disease-causing mutation was launched [110]. In this case, the first step relies on the introduction of a DSB in close proximity to the genomic site where the change is desired. This is made possible by using programmable designer nucleases, such as zinc finger nucleases (ZFNs), transcription activator-like effector nucleases (TALENs) or the recently introduced CRISPR-Cas (clustered regularly interspaced palindromic repeat-CRISPR-associated) systems. For a comprehensive description of the different types of designer nucleases, we refer the reader to detailed reviews published elsewhere [111,112,113]. As described in the previous sections, the cells have developed sophisticated mechanisms to sense and resolve DNA lesions, such as DSBs, in order to maintain genome integrity. The precise insertion of a DSB by such programmable nucleases triggers either of the two major repair mechanisms, NHEJ or HDR, which can be harnessed to achieve precise and permanent changes of the human genome. 

### 6.1. Use of NHEJ-Mediated Repair for Therapy

**Figure 3:**
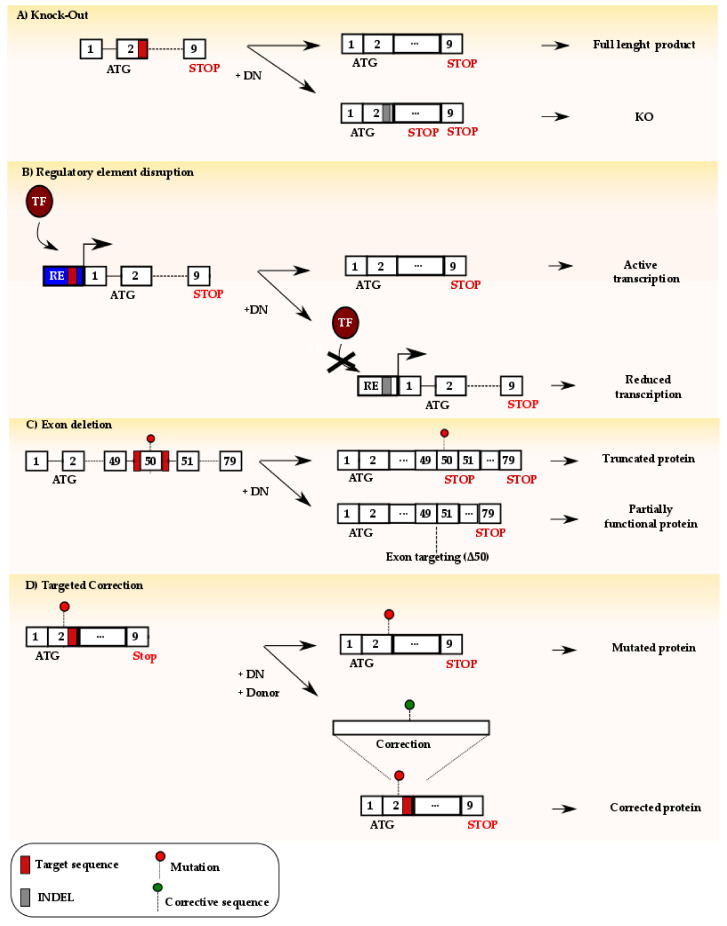
Genome editing using designer nucleases (DN): (**A**) Targeting of the DN to the coding region of a gene promotes the formation of a DSB which is typically repaired via NHEJ. The subsequent formation of indel mutations (grey box) may generate a premature stop codon, eventually leading to gene inactivation. (**B**) DN can be targeted to regulatory elements (RE) in order to disrupt the binding site of an activating transcription factor (TF). As a result, downstream gene transcription might be reduced or abolished in a cell-specific fashion as described in Section 6.1. (**C**) Two DNs can be directed to sites flanking an exon containing a non-sense mutation for its deletion. The resulting gene might result in the generation of a truncated, albeit partially functional, protein. (**D**) Targeting of a DN in proximity of a genetic mutation and simultaneous delivery of a donor template harboring the correct genetic sequence might result in precise gene editing via harnessing of the homology directed repair pathway.

In mammalian cells, NHEJ is the most commonly used pathway to repair a DSB. This mechanism is generally fast and promotes the ligation of the broken DNA without loss of genetic information in less than an hour [114]. However, the activity of the designer nucleases typically lasts for several hours, or longer. This results in the selection of repair events which lead to insertion or deletions of few nucleotides at the DSB site, the so-called indel mutations, that prevent further nuclease cleavage. This outcome has been used with the aim of inactivating endogenous genes or regulatory elements to provide a beneficial loss of function characteristic to patient cells (Figure 3A). The most well-documented example is the first clinically approved trial involving the use of designer nucleases to combat infections with the human immunodeficiency virus (HIV), which leads to a disease known as acquired immunodeficiency syndrome (AIDS). In this case, ZFNs have been used ex vivo to introduce indel mutations in the coding region of the *CCR5* gene in CD4+ T cells from HIV-positive patients (NCT00842634). This gene codes for the C-C chemokine receptor type 5, which is used as a co-receptor by HIV to infect host cells [115]. Its inactivation, as result of the ZFN-induced mutations, leads to acquired HIV-resistance, as documented by multiple studies [116]. Encouraging results have fostered further exploitation of this strategy in patient-derived hematopoietic stem cells with the goal of providing transplanted patients with a new immune system that is resistant to the virus (NCT02500849) [117]. Similarly, the targeted introduction of indel mutations can be explored to inactivate regulatory elements (Figure 3B). Beta-hemoglobinopathies include a set of different disorders caused by mutations in the *HBB* gene that result in impaired oxygenation of organ tissues leading to multi-organ failure and death. IVSI-110 G>A is a common intronic mutation in the β-globin encoded by the *HBB* gene. The point mutation leads to the formation of an aberrant splice acceptor that drives abnormal splicing and hence nonfunctional β-globin protein [118]. In the attempt to restore normal splicing, both CRISPR-Cas and TALENs have been used to introduce indel mutations to disrupt the aberrant splice site. The high efficiency of this approach and the large number of mutations that could be treated using such strategies are promising to prompt future investigation [119]. Recently, genome editing for the treatment of beta-hemoglobinopathies has entered clinics with two ongoing trials. Reactivation of fetal hemoglobin expression holds promise to complement the adult hemoglobin function [120]. In both clinical studies, the goal is to reactivate the expression of the fetal hemoglobin by inhibiting the expression of the *BCL11A* (B cell lymphoma/leukemia 11A) gene using either ZFN or CRISPR-Cas (NCT03432364 and NCT03655678, respectively). This gene encodes for a critical transcription factor that regulates the switch between fetal and adult hemoglobin [121], acting as a repressor of the former. Preclinical studies have shown that the disruption of the erythroid-specific enhancer of the *BCL11A* gene leads to its reduced expression and to the reactivation of fetal hemoglobin to therapeutic levels [122]. The results of these clinical studies will be certainly instrumental to further develop new therapeutics for patient affected by hemoglobin disorders. The therapeutic potential of targeted indels has been explored also to mitigate one of the most devastating human genetic disorders. Duchenne muscular dystrophy (DMD) is a deadly X-linked disease resulting from mutations in the *dystrophin* gene. Dystrophin is a structural protein that connects actin fibers and cytoskeleton to the muscle fibers via a rod-shaped domain. In its absence, muscle cells progressively degenerate, resulting in slow but constant inflammation and fibrosis, which eventually leads to death due to respiratory insufficiency or heart failure in early adulthood [123]. Different mutations have been described that result in premature stop codons leading to a lack of the dystrophin protein. Since dystrophin is a structural protein, amino acid substitution or truncations within the rod domain are not necessarily critical for its function. This has prompted the development of new strategies to create mutations that eliminate the stop codon, thus restoring the reading frame of the protein [124]. Considering the stochastic nature of this approach, only a third of the mutations result in dystrophin expression. Therefore, to increase the likelihood of creating indels that restore protein expression, researchers have explored alternative strategies. Introducing a DSB in between regions of microhomology leads to its repair preferentially via the MMEJ pathway that, as explained in Section 4.5.3, results in the formation of precise microdeletions. MMEJ has been exploited to precisely correct frameshift mutations resulting from microduplications in patient-derived cells for diverse rare diseases without the need for additional donor templates [125]. Recently, a bioinformatics tool has been described that allows the identification of targets which are amenable for MMEJ repair, further promoting the possibility of achieving accurate repair in an HDR-independent way [126]. This will certainly provide in the future new exciting opportunities for preclinical exploitation of DMD therapeutics based on genome editing. In a different approach, using two designer nucleases targeting sites flanking a mutation-bearing exon led to its deletion with consequent reconstitution of dystrophin expression (Figure 3C) [124]. In both cases, the expression of a new dystrophin harboring a shorter rod domain resulted in a milder form of DMD similar to Becker-muscular dystrophy (BMD), with patients having typically a prolonged life expectancy if the heart muscle is not affected [127]. However, immunological concerns related to the expression of novel epitopes due to restored dystrophin expression must be considered [128]. 

In conclusion, the predominant use of NHEJ and the newly explored MMEJ in mammalian cells enable the highly efficient introduction of loss of function mutations with allelic frequencies as high as 90% in some instances [129]. This high efficacy supports the development of further strategies exploiting this mechanism for other therapeutic purposes. 

### 6.2. Use of HDR-Mediated Repair for Therapy

In some instances, the stochastic occurrence of indel mutations upon NHEJ-mediated DSB repair is not sufficient to secure a therapeutic benefit. In such cases, strategies aimed at harnessing the HDR mechanism to achieve precise editing at the DSB site are paramount. Two major challenges have to be considered in this context: (i) a typical human cell rather engages NHEJ than HDR to repair a DSB, and (ii) the edited gene can be cleaved again by the designer nuclease in certain circumstances. In the 1990s, pioneer studies from Maria Jasin’s lab have shown that the introduction of a targeted DSB and the simultaneous delivery of an exogenous DNA fragment that is homologous to the target site improve the efficiency of HDR by several orders of magnitude [130]. The goal of biasing the DNA repair towards HDR has been pursued by several laboratories worldwide with different strategies. These include the use of compounds to either synchronize the cells or to restrict the expression of the designer nuclease in the cell cycle phases that support HDR [131]. Similarly, the use of small molecule drugs to either directly inhibit NHEJ or to boost HDR has been explored [132,133]. While promising, such approaches rely on global alterations of cell physiology, with potential deleterious effects on key mechanism that rely on NHEJ or HDR, with devastating consequences as discussed in Section 5.1. A localized approach in which the increase of HDR is restricted to the target site would be preferable. Hence, direct fusion of the Cas9 endonuclease to proteins that inhibit NHEJ or promote HDR at the target site, such as dominant negative forms of 53BP1 or CtIP, respectively, have been explored [134,135]. The knowledge acquired in the last decades has fueled the development of precise genome editing strategies to treat human genetic disorders. Even though current clinical trial explore the harnessing of NHEJ-based correction to treat beta-hemoglobinopathies [136], as described in the previous paragraph, this group of disorders is a particularly fit candidate for the development of precise genome editing therapeutics (Figure 3D). In this context, it has been indeed estimated that gene correction frequencies as low as 2% in hematopoietic stem cells might result in therapeutic benefit when edited cells are transplanted back to sick patients [137]. Different gene-correction strategies have been pursued with promising success to correct the adenine to thymidine mutation that eventually leads to the production of sickling hemoglobin and to the development of sickle cell disease (SCD) [138]. The development of protocols capable of isolating edited stem cells which retain engraftment potential suggests the great potential that precise genome editing strategies have for curing devastating disorders such as SCD [139]. Similarly, also primary immunodeficiencies (PIDs) are considered a suitable candidate for therapeutic strategies based on precise genome editing. A successful example is the development of genome editing strategies to treat X-linked chronic granulomatous disease (X-CGD) caused by mutations in the *CYBB* gene. In this case, either the correction of the *CYBB* causative mutation [140] or the integration of a *CYBB* expression cassette in a “safe harbor” locus to complement the missing gene function has been pursued [141]. The results achieved so far underline that precise genome editing is certainly an opportunity for patients, and further studies will be required to dissect its potential in the clinics.

## 7. Concluding Remarks

Understanding how genome stability is maintained and investigating the mechanisms adopted by the cells to withstand DNA lesions has been paramount to explore the therapeutic potential of genome editing. We have illustrated how failure in DNA repair mechanisms may contribute to the onset of detrimental conditions, such as cancer. Moreover, we have described how this knowledge is exploited to develop new therapeutics based on “synthetic lethality” or genome editing using designer nucleases. While the activity of designer nucleases at sites that share a certain sequence identity with the target site, the so-called off-targets, poses concerns [142], the increasing number of genome editing trials approved thus far [143] and the first in human application of CRISPR-Cas to treat a blindness disorder (NCT03872479) recently described suggest that new therapeutics are on the horizon. Once current approaches are substantiated in conditions that better resemble those of transplanted patients and genome-wide analysis to dissect the genotoxic potential of these approaches are in place, it will be reasonable to believe that new treatment opportunities will be available for more and more human disorders using a new generation of therapeutics.

## References

[1] Jackson S.P., Bartek J. (2009). The DNA-damage response in human biology and disease. Nature.

[2] Lindahl T., Barnes D. (2000). Repair of endogenous DNA damage. Cold Spring Harb. Symp. Quant. Boil..

[3] Ganai R.A., Johansson E. (2016). DNA Replication—A Matter of Fidelity. Mol. Cell.

[4] Cadet J., Wagner J.R. (2013). DNA Base Damage by Reactive Oxygen Species, Oxidizing Agents, and UV Radiation. Cold Spring Harb. Perspect. Boil..

[5] Van Houten B., Santa-Gonzalez G.A., Camargo M. (2017). DNA repair after oxidative stress: Current challenges. Curr. Opin. Toxicol..

[6] Chatterjee N., Walker G.C. (2017). Mechanisms of DNA damage, repair, and mutagenesis. Environ. Mol. Mutagen..

[7] Tubbs A., Nussenzweig A. (2017). Endogenous DNA Damage as a Source of Genomic Instability in Cancer. Cell.

[8] Lindahl T. (1993). Instability and decay of the primary structure of DNA. Nature.

[9] Hossain A., Lin Y., Yan S. (2018). Single-Strand Break End Resection in Genome Integrity: Mechanism and Regulation by APE2. Int. J. Mol. Sci..

[10] Abbotts R., Wilson D.M. (2017). Coordination of DNA single strand break repair. Free. Radic. Boil. Med..

[11] Callen E., Zong D., Wu W., Wong N., Stanlie A., Ishikawa M., Pavani R., Dumitrache L.C., Byrum A.K., Mendez-Dorantes C. (2020). 53BP1 Enforces Distinct Pre- and Post-resection Blocks on Homologous Recombination. Mol. Cell.

[12] Li Z., Pearlman A.H., Hsieh P. (2016). DNA mismatch repair and the DNA damage response. DNA Repair.

[13] Wang K., Li L., Zhang Y., Gao D. (2019). Crosstalk between signaling pathways and DNA damage response. Genome Instab. Dis..

[14] Martinez D.L., Liang C.-C., Cohn M.A. (2016). Cellular response to DNA interstrand crosslinks: The Fanconi anemia pathway. Cell. Mol. Life Sci..

[15] Motnenko A., Liang C.-C., Yang D., Martinez D.L., Yoshikawa Y., Zhan B., Ward K.E., Tian J., Haas W., Spingardi P. (2018). Identification of UHRF2 as a novel DNA interstrand crosslink sensor protein. PLoS Genet..

[16] Eustermann S., Wu W.-F., Langelier M.-F., Yang J.-C., Easton L.E., Riccio A.A., Pascal J.M., Neuhaus D. (2015). Structural Basis of Detection and Signaling of DNA Single-Strand Breaks by Human PARP-1. Mol. Cell.

[17] Blackford A.N., Jackson S.P. (2017). ATM, ATR, and DNA-PK: The Trinity at the Heart of the DNA Damage Response. Mol. Cell.

[18] Alexander J.L., Orr-Weaver T.L. (2016). Replication fork instability and the consequences of fork collisions from rereplication. Genes Dev..

[19] Reginato G., Cejka P. (2020). The MRE11 complex: A versatile toolkit for the repair of broken DNA. DNA Repair.

[20] Polo S., Jackson S.P. (2011). Dynamics of DNA damage response proteins at DNA breaks: A focus on protein modifications. Genes Dev..

[21] Awasthi P., Foiani M., Kumar A. (2015). ATM and ATR signaling at a glance. J. Cell Sci..

[22] Chen J. (2016). The Cell-Cycle Arrest and Apoptotic Functions of p53 in Tumor Initiation and Progression. Cold Spring Harb. Perspect. Med..

[23] Shaltiel I.A., Krenning L., Bruinsma W., Medema R. (2015). The same, only different—DNA damage checkpoints and their reversal throughout the cell cycle. J. Cell Sci..

[24] Houtgraaf J.H., Versmissen J., Van Der Giessen W.J. (2006). A concise review of DNA damage checkpoints and repair in mammalian cells. Cardiovasc. Revascularization Med..

[25] Lim S., Kaldis P. (2013). Cdks, cyclins and CKIs: Roles beyond cell cycle regulation. Development.

[26] Trimarchi J.M., Lees J.A. (2002). Sibling rivalry in the E2F family. Nat. Rev. Mol. Cell Boil..

[27] Harbour J.W., Dean D.C. (2000). The Rb/E2F pathway: Expanding roles and emerging paradigms. Genes Dev..

[28] Ding L., Cao J., Lin W., Chen H., Xiong X., Ao H., Yu M., Lin J., Cui Q. (2020). The Roles of Cyclin-Dependent Kinases in Cell-Cycle Progression and Therapeutic Strategies in Human Breast Cancer. Int. J. Mol. Sci..

[29] Petr M.A., Tulika T., Carmona-Marin L.M., Scheibye-Knudsen M. (2020). Protecting the Aging Genome. Trends Cell Boil..

[30] Wang J.Y.J. (2019). Cell Death Response to DNA Damage. Yalej. Biol. Med..

[31] Fischer M., Quaas M., Nickel A., Engeland K. (2015). Indirect p53-dependent transcriptional repression of Survivin, CDC25C, and PLK1 genes requires the cyclin-dependent kinase inhibitor p21/CDKN1A and CDE/CHR promoter sites binding the DREAM complex. Oncotarget.

[32] Thornton T.M. (2009). Non-Classical P38 Map Kinase Functions: Cell Cycle Checkpoints and Survival. Int. J. Boil. Sci..

[33] Raman M., Earnest S., Zhang K., Zhao Y., Cobb M.H. (2007). TAO kinases mediate activation of p38 in response to DNA damage. Embo J..

[34] Reinhardt H.C., Aslanian A.S., Lees J.A., Yaffe M.B. (2007). p53-Deficient Cells Rely on ATM- and ATR-Mediated Checkpoint Signaling through the p38MAPK/MK2 Pathway for Survival after DNA Damage. Cancer Cell.

[35] Kim G.-Y., Mercer S.E., Ewton D.Z., Yan Z., Jin K., Friedman E. (2002). The Stress-activated Protein Kinases p38α and JNK1 Stabilize p21Cip1 by Phosphorylation. J. Boil. Chem..

[36] Barr A.R., Cooper S., Heldt F.S., Butera F., Stoy H., Mansfeld J., Novak B., Bakal C. (2017). DNA damage during S-phase mediates the proliferation-quiescence decision in the subsequent G1 via p21 expression. Nat. Commun..

[37] Rhind N., Russell P. (2001). Checkpoints: It takes more than time to heal some wounds. Curr. Boil..

[38] Bartek J., Lukas C., Lukas J. (2004). Checking on DNA damage in S phase. Nat. Rev. Mol. Cell Boil..

[39] Yekezare M., Gómez-González B., Diffley J.F.X. (2013). Controlling DNA replication origins in response to DNA damage–inhibit globally, activate locally. J. Cell Sci..

[40] Gire V., Dulić V. (2015). Senescence from G2 arrest, revisited. Cell Cycle.

[41] Bennett G., Papamichos-Chronakis E., Peterson C.L. (2013). DNA repair choice defines a common pathway for recruitment of chromatin regulators. Nat. Commun..

[42] Deans A.J., West S.C. (2011). DNA interstrand crosslink repair and cancer. Nat. Rev. Cancer.

[43] Douwel D.K., Boonen R.A., Long D., Szypowska A.A., Räschle M., Walter J.C., Knipscheer P. (2014). XPF-ERCC1 acts in unhooking DNA interstrand crosslinks in cooperation with FANCD2 and FANCP/SLX4. Mol. Cell.

[44] Puumalainen M.-R., Rüthemann P., Min J.-H., Naegeli H. (2015). Xeroderma pigmentosum group C sensor: Unprecedented recognition strategy and tight spatiotemporal regulation. Cell. Mol. Life Sci..

[45] Spivak G. (2015). Nucleotide excision repair in humans. DNA Repair.

[46] Schärer O.D. (2013). Nucleotide Excision Repair in Eukaryotes. Cold Spring Harb. Perspect. Boil..

[47] Krokan H.E., Bjørås M. (2013). Base excision repair. Cold Spring Harb. Perspect. Biol..

[48] Freudenthal B.D. (2017). Base excision repair of oxidative DNA damage from mechanism to disease. Front. Biosci..

[49] Erie D.A., Weninger K.R. (2014). Single molecule studies of DNA mismatch repair. DNA Repair.

[50] Hsieh P., Zhang Y. (2017). The Devil is in the details for DNA mismatch repair. Proc. Natl. Acad. Sci. USA.

[51] Scully R., Panday A., Elango R., Willis N.A. (2019). DNA double-strand break repair-pathway choice in somatic mammalian cells. Nat. Rev. Mol. Cell Boil..

[52] Marini F., Rawal C.C., Liberi G., Pellicioli A. (2019). Regulation of DNA Double Strand Breaks Processing: Focus on Barriers. Front. Mol. Biosci..

[53] Chang H.H.Y., Pannunzio N.R., Adachi N., Lieber M.R. (2017). Non-homologous DNA end joining and alternative pathways to double-strand break repair. Nat. Rev. Mol. Cell Boil..

[54] Ahnesorg P., Smith P., Jackson S.P. (2006). XLF Interacts with the XRCC4-DNA Ligase IV Complex to Promote DNA Nonhomologous End-Joining. Cell.

[55] Brouwer I., Sitters G., Candelli A., Heerema S.J., Heller I., De A.J.M., Zhang H., Normanno D., Modesti M., Peterman E.J. (2016). Sliding sleeves of XRCC4–XLF bridge DNA and connect fragments of broken DNA. Nature.

[56] Wu Q. (2019). Structural mechanism of DNA-end synapsis in the non-homologous end joining pathway for repairing double-strand breaks: Bridge over troubled ends. Biochem. Soc. Trans..

[57] Goodarzi A.A., Yu Y., Riballo E., Douglas P., Walker S., Ye R., Härer C., Marchetti C., Morrice N., Jeggo P.A. (2006). DNA-PK autophosphorylation facilitates Artemis endonuclease activity. Embo J..

[58] Ochi T., Blackford A.N., Coates J., Jhujh S., Mehmood S., Tamura N., Travers J., Wu Q., Draviam V.M., Robinson C.V. (2015). PAXX, a paralog of XRCC4 and XLF, interacts with Ku to promote DNA double-strand break repair. Science.

[59] Weinfeld M., Mani R.S., Abdou I., Aceytuno R.D., Glover J.M., Acetuno R.D. (2011). Tidying up loose ends: The role of polynucleotide kinase/phosphatase in DNA strand break repair. Trends Biochem. Sci..

[60] Chang H.H.Y., Watanabe G., Gerodimos C.A., Ochi T., Blundell T.L., Jackson S.P., Lieber M.R. (2016). Different DNA End Configurations Dictate Which NHEJ Components Are Most Important for Joining Efficiency. J. Boil. Chem..

[61] Cui X., Yu Y., Gupta S., Cho Y.-M., Lees-Miller S.P., Meek K. (2005). Autophosphorylation of DNA-Dependent Protein Kinase Regulates DNA End Processing and May Also Alter Double-Strand Break Repair Pathway Choice. Mol. Cell. Boil..

[62] Fumagalli M., Rossiello F., Mondello C., Di Fagagna F.D. (2014). Stable Cellular Senescence Is Associated with Persistent DDR Activation. PLoS ONE.

[63] Roos W.P., Thomas A.D., Kaina B. (2015). DNA damage and the balance between survival and death in cancer biology. Nat. Rev. Cancer.

[64] Syed A., A Tainer J. (2018). The MRE11–RAD50–NBS1 Complex Conducts the Orchestration of Damage Signaling and Outcomes to Stress in DNA Replication and Repair. Annu. Rev. Biochem..

[65] Myler L.R., Gallardo I.F., Soniat M.M., Deshpande R.A., Gonzalez X.B., Kim Y., Paull T.T., Finkelstein I.J. (2017). Single-Molecule Imaging Reveals How Mre11-Rad50-Nbs1 Initiates DNA Break Repair. Mol. Cell.

[66] Jasin M., Rothstein R. (2013). Repair of Strand Breaks by Homologous Recombination. Cold Spring Harb. Perspect. Boil..

[67] Hopfner K.-P., Craig L., Moncalián G., Zinkel R.A., Usui T., Owen B.A.L., Karcher A., Henderson B., Bodmer J.-L., McMurray C.T. (2002). The Rad50 zinc-hook is a structure joining Mre11 complexes in DNA recombination and repair. Nature.

[68] Garcia V., Phelps S.E.L., Gray S., Neale M.J. (2011). Bidirectional resection of DNA double-strand breaks by Mre11 and Exo1. Nature.

[69] Langerak P., Mejia-Ramirez E., Limbo O., Russell P. (2011). Release of Ku and MRN from DNA Ends by Mre11 Nuclease Activity and Ctp1 Is Required for Homologous Recombination Repair of Double-Strand Breaks. PLoS Genet..

[70] Wang H., Shi L.Z., Wong C.C., Han X., Hwang P.Y.-H., Truong L.N., Zhu Q., Shao Z., Chen D.J., Berns M.W. (2013). The Interaction of CtIP and Nbs1 Connects CDK and ATM to Regulate HR–Mediated Double-Strand Break Repair. PLoS Genet..

[71] Daley J.M., Jimenez-Sainz J., Wang W., Miller A.S., Xue X., Nguyen K.A., Jensen R.B., Sung P. (2017). Enhancement of BLM-DNA2-Mediated Long-Range DNA End Resection by CtIP. Cell Rep..

[72] Guirouilh-Barbat J., Gelot C., Xie A., Dardillac E., Scully R., Lopez B.S. (2016). 53BP1 Protects against CtIP-Dependent Capture of Ectopic Chromosomal Sequences at the Junction of Distant Double-Strand Breaks. PLoS Genet..

[73] Tiwari A., Jones O.A., Chan K.-L. (2018). 53BP1 can limit sister-chromatid rupture and rearrangements driven by a distinct ultrafine DNA bridging-breakage process. Nat. Commun..

[74] Misenko S.M., Patel D.S., Her J., Bunting S.F. (2018). DNA repair and cell cycle checkpoint defects in a mouse model of ‘BRCAness’ are partially rescued by 53BP1 deletion. Cell Cycle.

[75] Yates L., Aramayo R.J., Pokhrel N., Caldwell C., Kaplan J.A., Perera R., Spies M., Antony E., Zhang X. (2018). A structural and dynamic model for the assembly of Replication Protein A on single-stranded DNA. Nat. Commun..

[76] Deng S.K., Chen H., Symington L.S. (2014). Replication protein A prevents promiscuous annealing between short sequence homologies: Implications for genome integrity. BioEssays.

[77] Ma C.J., Gibb B., Kwon Y., Sung P., Greene E.C. (2016). Protein dynamics of human RPA and RAD51 on ssDNA during assembly and disassembly of the RAD51 filament. Nucleic Acids Res..

[78] Godin S.K., Sullivan M.R., Bernstein K.A. (2016). Novel insights into RAD51 activity and regulation during homologous recombination and DNA replication. Biochem. Cell Boil..

[79] Van Der Heijden T., Modesti M., Hage S., Kanaar R., Wyman C., Dekker C. (2008). Homologous Recombination in Real Time: DNA Strand Exchange by RecA. Mol. Cell.

[80] Wright W.D., Shah S.S., Heyer W.-D. (2018). Homologous recombination and the repair of DNA double-strand breaks. J. Boil. Chem..

[81] Sallmyr A., Tomkinson A.E. (2018). Repair of DNA double-strand breaks by mammalian alternative end-joining pathways. J. Boil. Chem..

[82] Ceccaldi R., Rondinelli B., D’Andrea A. (2015). Repair Pathway Choices and Consequences at the Double-Strand Break. Trends Cell Boil..

[83] Pon J.R., Marra M.A. (2015). Driver and Passenger Mutations in Cancer. Annu. Rev. Pathol. Mech. Dis..

[84] Rogozin I.B., Goncearenco A., Lada A.G., De S., Yurchenko V., Nudelman G., Panchenko A.R., Cooper D.N., Pavlov Y.V. (2018). DNA polymerase η mutational signatures are found in a variety of different types of cancer. Cell Cycle.

[85] Abdel-Rahman M.H., Sample K.M., Pilarski R., Walsh T., Grosel T., Kinnamon D., Boru G., Massengill J.B., Schoenfield L., Kelly B. (2020). Whole Exome Sequencing Identifies Candidate Genes Associated with Hereditary Predisposition to Uveal Melanoma. Ophthalmology.

[86] Tiwari V., Wilson D.M. (2019). DNA Damage and Associated DNA Repair Defects in Disease and Premature Aging. Am. J. Hum. Genet..

[87] Woodbine L., Gennery A.R., Jeggo P.A. (2014). The clinical impact of deficiency in DNA non-homologous end-joining. DNA Repair.

[88] Chi X., Li Y., Qiu X. (2020). V(D)J recombination, somatic hypermutation and class switch recombination of immunoglobulins: Mechanism and regulation. Immunology.

[89] Mansilla-Soto J., Cortes P. (2003). VDJ recombination: Artemis and its in vivo role in hairpin opening. J. Exp. Med..

[90] Justiz Vaillant A.A., Mohseni M. (2019). Severe Combined Immunodeficiency.

[91] Mersch J., Jackson M.A., Park M., Nebgen D.R., Peterson S.K., Singletary C.N., Arun B.K., Litton J.K. (2014). Cancers associated with BRCA1 and BRCA2 mutations other than breast and ovarian. Cancer.

[92] Noh J.M., Choi D.H., Baek H., Nam S.J., Lee J.E., Kim J.W., Ki C.-S., Park W., Huh S.J. (2012). Associations betweenBRCAMutations in High-Risk Breast Cancer Patients and Familial Cancers Other than Breast or Ovary. J. Breast Cancer.

[93] Buisson R., Niraj J., Rodrigue A., Ho C.K., Kreuzer J., Foo T.K., Hardy E.J.-L., Dellaire G., Haas W., Xia B. (2017). Coupling of Homologous Recombination and the Checkpoint by ATR. Mol. Cell.

[94] Macedo G.S., Alemar B., Ashton-Prolla P. (2019). Reviewing the characteristics of BRCA and PALB2-related cancers in the precision medicine era. Genet. Mol. Boil..

[95] Soria-Bretones I., Sáez C., Ruíz-Borrego M., Japón M., Huertas P. (2013). Prognostic value of CtIP/RBBP8 expression in breast cancer. Cancer Med..

[96] Luengo A., Gui D.Y., Heiden M.G.V. (2017). Targeting Metabolism for Cancer Therapy. Cell Chem. Boil..

[97] Delgado J.L., Hsieh C.-M., Chan N.-L., Hiasa H. (2018). Topoisomerases as anticancer targets. Biochem. J..

[98] Lord C.J., Tutt A.N., Ashworth A. (2015). Synthetic Lethality and Cancer Therapy: Lessons Learned from the Development of PARP Inhibitors. Annu. Rev. Med..

[99] Wang Y., Xia Y., Lu Z. (2018). Metabolic features of cancer cells. Cancer Commun..

[100] Jaiswal A.S., Banerjee S., Aneja R., Sarkar F.H., Ostrov D.A., Narayan S. (2011). DNA Polymerase β as a Novel Target for Chemotherapeutic Intervention of Colorectal Cancer. PLoS ONE.

[101] Barakat. K., Gajewski M.M., Tuszynski J.A. (2012). DNA polymerase beta (pol β) inhibitors: A comprehensive overview. Drug Discov. Today.

[102] Nickoloff J.A., Jones D., Lee S.-H., Williamson E.A., Hromas R. (2017). Drugging the Cancers Addicted to DNA Repair. J. Natl. Cancer Inst..

[103] Chen C.-C., Feng W., Lim P.X., Kass E.M., Jasin M. (2017). Homology-Directed Repair and the Role of BRCA1, BRCA2, and Related Proteins in Genome Integrity and Cancer. Annu. Rev. Cancer Boil..

[104] Palmbos P.L., Hussain M.H. (2016). Targeting PARP in Prostate Cancer: Novelty, Pitfalls, and Promise. Oncol. (Williston Park. N.Y.).

[105] Mirza M., Pignata S., Ledermann J. (2018). Latest clinical evidence and further development of PARP inhibitors in ovarian cancer. Ann. Oncol..

[106] Rottenberg S., Jaspers J.E., Kersbergen A., Van Der Burg E., Nygren A.O.H., Zander S.A.L., Derksen P.W.B., De Bruin M., Zevenhoven J., Lau A. (2008). High sensitivity of BRCA1-deficient mammary tumors to the PARP inhibitor AZD2281 alone and in combination with platinum drugs. Proc. Natl. Acad. Sci. USA.

[107] Konstantinopoulos P.A., Waggoner S., Vidal G.A., Mita M., Moroney J.W., Holloway R., Van Le L., Sachdev J.C., Chapman-Davis E., Colon-Otero G. (2019). Single-Arm Phases 1 and 2 Trial of Niraparib in Combination With Pembrolizumab in Patients With Recurrent Platinum-Resistant Ovarian Carcinoma. JAMA Oncol..

[108] Gornstein E.L., Sandefur S., Chung J.H., Gay L.M., Holmes O., Erlich R.L., Soman S., Martin L.K., Rose A.V., Stephens P.J. (2018). BRCA2 Reversion Mutation Associated With Acquired Resistance to Olaparib in Estrogen Receptor-positive Breast Cancer Detected by Genomic Profiling of Tissue and Liquid Biopsy. Clin. Breast Cancer.

[109] Naldini L., Trono D., Verma I.M. (2016). Lentiviral vectors, two decades later. Science.

[110] Doudna J.A. (2020). The promise and challenge of therapeutic genome editing. Nature.

[111] Carroll D. (2011). Genome Engineering with Zinc-Finger Nucleases. Genetics.

[112] Mussolino C., Cathomen T. (2012). TALE nucleases: Tailored genome engineering made easy. Curr. Opin. Biotechnol..

[113] Mussolino C., Cathomen T. (2013). RNA guides genome engineering. Nat. Biotechnol..

[114] Mao Z., Bozzella M., Seluanov A., Gorbunova V. (2008). Comparison of nonhomologous end joining and homologous recombination in human cells. DNA Repair.

[115] Lederman M.M., Penn-Nicholson A., Cho M., Mosier N. (2006). Biology of CCR5 and Its Role in HIV Infection and Treatment. JAMA.

[116] Cornu T.I., Mussolino C., Bloom K., Cathomen T. (2015). Editing CCR5: A Novel Approach to HIV Gene Therapy. Adv. Exp. Med. Biol..

[117] Tebas P., Stein D., Tang W.W., Frank I., Wang S.Q., Lee G., Spratt S.K., Surosky R.T., Giedlin M.A., Nichol G. (2014). Gene editing of CCR5 in autologous CD4 T cells of persons infected with HIV. N. Engl. J. Med..

[118] Patsali P., Turchiano G., Papasavva P., Romito M., Loucari C.C., Stephanou C., Christou S., Sitarou M., Mussolino C., Cornu T.I. (2019). Correction of IVS I-110(G>A) β-thalassemia by CRISPR/Cas-and TALEN-mediated disruption of aberrant regulatory elements in human hematopoietic stem and progenitor cells. Haematologica.

[119] Patsali P., Mussolino C., Ladas P., Floga A., Kolnagou A., Christou S., Sitarou M., Antoniou M.N., Cathomen T., Lederer C.W. (2019). The Scope for Thalassemia Gene Therapy by Disruption of Aberrant Regulatory Elements. J. Clin. Med..

[120] Sankaran V.G., Orkin S.H. (2012). The Switch from Fetal to Adult Hemoglobin. Cold Spring Harb. Perspect. Med..

[121] Sankaran V.G., Menne T.F., Xu J., Akie T.E., Lettre G., Van Handel B., Mikkola H.K.A., Hirschhorn J.N., Cantor A.B., Orkin S.H. (2008). Human Fetal Hemoglobin Expression Is Regulated by the Developmental Stage-Specific Repressor BCL11A. Science.

[122] Psatha N., Reik A., Phelps S., Zhou Y., Dalas D., Yannaki E., Levasseur D.N., Urnov F.D., Holmes M.C., Papayannopoulou T. (2018). Disruption of the BCL11A Erythroid Enhancer Reactivates Fetal Hemoglobin in Erythroid Cells of Patients with β-Thalassemia Major. Mol. Ther. Methods Clin. Dev..

[123] Salmaninejad A., Valilou S.F., Bayat H., Ebadi N., Daraei A., Yousefi M., Nesaei A., Mojarrad M. (2018). Duchenne muscular dystrophy: An updated review of common available therapies. Int. J. Neurosci..

[124] Ousterout D.G., Perez-Pinera P., Thakore P.I., Kabadi A.M., Brown M.T., Qin X., Fedrigo O., Mouly V., Tremblay J.P., Gersbach A.C. (2013). Reading Frame Correction by Targeted Genome Editing Restores Dystrophin Expression in Cells From Duchenne Muscular Dystrophy Patients. Mol. Ther..

[125] Iyer S., Suresh S., Guo D., Daman K., Chen J.C.J., Liu P., Zieger M., Luk K., Roscoe B.P., Mueller C. (2019). Precise therapeutic gene correction by a simple nuclease-induced double-stranded break. Nature.

[126] Grajcarek J., Monlong J., Nishinaka-Arai Y., Nakamura M., Nagai M., Matsuo S., Lougheed D., Sakurai H., Saito M., Bourque G. (2019). Genome-wide microhomologies enable precise template-free editing of biologically relevant deletion mutations. Nat. Commun..

[127] Vengalil S., Preethish-Kumar V., Polavarapu K., Mahadevappa M., Sekar D., Purushottam M., Thomas P.T., Nashi S., Nalini A. (2017). Duchenne Muscular Dystrophy and Becker Muscular Dystrophy Confirmed by Multiplex Ligation-Dependent Probe Amplification: Genotype-Phenotype Correlation in a Large Cohort. J. Clin. Neurol..

[128] Mendell J.R., Campbell K., Rodino-Klapac L., Sahenk Z., Shilling C., Lewis S., Bowles D., Gray S., Li C., Galloway G. (2010). Dystrophin immunity in Duchenne’s muscular dystrophy. N. Engl. J. Med..

[129] Gundry M., Brunetti L., Lin A., Mayle A., Kitano A., Wagner D.L., Hsu J.I., Hoegenauer K.A., Rooney C.M., Goodell M.A. (2016). Highly Efficient Genome Editing of Murine and Human Hematopoietic Progenitor Cells by CRISPR/Cas9. Cell Rep..

[130] Rouet P., Smih F., Jasin M. (1994). Expression of a site-specific endonuclease stimulates homologous recombination in mammalian cells. Proc. Natl. Acad. Sci. USA.

[131] Lin S., Staahl B.T., Alla R.K., Doudna J.A. (2014). Enhanced homology-directed human genome engineering by controlled timing of CRISPR/Cas9 delivery. eLife.

[132] Song J., Yang D., Xu J., Zhu T., Chen Y.E., Zhang J. (2016). RS-1 enhances CRISPR/Cas9- and TALEN-mediated knock-in efficiency. Nat. Commun..

[133] Hu Z., Shi Z., Guo X., Jiang B., Wang G., Luo D., Chen Y., Zhu Y.-S. (2018). Ligase IV inhibitor SCR7 enhances gene editing directed by CRISPR–Cas9 and ssODN in human cancer cells. Cell Biosci..

[134] Charpentier M., Khedher A.H.Y., Menoret S., Brion A., Lamribet K., Dardillac E., Boix C., Perrouault L., Tesson L., Geny S. (2018). CtIP fusion to Cas9 enhances transgene integration by homology-dependent repair. Nat. Commun..

[135] Paulsen B.S., Mandal P.K., Frock R.L., Boyraz B., Yadav R., Upadhyayula S., Gutierrez-Martinez P., Ebina W., Fasth A., Kirchhausen T. (2017). Ectopic expression of RAD52 and dn53BP1 improves homology-directed repair during CRISPR-Cas9 genome editing. Nat. Biomed. Eng..

[136] Canver M., Orkin S.H. (2016). Customizing the genome as therapy for the β-hemoglobinopathies. Blood.

[137] Porteus M.H., Malik P., Tisdale J. (2017). Genome Editing for the β-Hemoglobinopathies BT-Gene and Cell Therapies for Beta-Globinopathies.

[138] Dever D.P., Porteus M. (2017). The changing landscape of gene editing in hematopoietic stem cells. Curr. Opin. Hematol..

[139] Agudelo D., Duringer A., Bozoyan L., Huard C.C., Carter S., Loehr J., Synodinou D., Drouin M., Salsman J., Dellaire G. (2017). Marker-free coselection for CRISPR-driven genome editing in human cells. Nat. Methods.

[140] De Ravin S.S., Li L., Wu X., Choi U., Allen C., Koontz S., Lee J., Theobald-Whiting N., Chu J., Garofalo M. (2017). CRISPR-Cas9 gene repair of hematopoietic stem cells from patients with X-linked chronic granulomatous disease. Sci. Transl. Med..

[141] De Ravin S.S., Reik A., Liu P.-Q., Li L., Wu X., Su L., Raley C., Theobald N., Choi U., Song A.H. (2016). Targeted gene addition in human CD34+ hematopoietic cells for correction of X-linked chronic granulomatous disease. Nat. Biotechnol..

[142] Yee J.-K. (2016). Off-target effects of engineered nucleases. FEBS J..

[143] Hirakawa M.P., Krishnakumar R., Timlin J.A., Carney J.P., Butler K.S. (2020). Gene editing and CRISPR in the clinic: Current and future perspectives. Biosci. Rep..

